# Perceptions of the family physician from adolescents and their caregivers preparing to transition to adult care

**DOI:** 10.1186/s12875-018-0830-6

**Published:** 2018-08-23

**Authors:** Angela Xiao Han, Sandy Rosalie Whitehouse, Steve Tsai, Sandy Hwang, Sally Thorne

**Affiliations:** 1 University of Alberta, 116 St & 85 Ave, Edmonton, AB T6G 2R3 Canada; 2 University of British Columbia, 2329 West Mall, Vancouver, BC V6T 1Z4 Canada; 3 Child Family Research Institute, 950 W 28th Ave, Vancouver, BC V5Z 4H4 Canada; 4 University of British Columbia School of Nursing, T201-2211 Wesbrook Mall, Vancouver, BC V6T 2B5 Canada

**Keywords:** Adolescent, Chronic illness, Family physician, Medical home, Transition

## Abstract

**Background:**

Adolescents with chronic health conditions and/or disabilities (CHC/D’s) often face challenges when transitioning to adult care, which leads to a higher risk of morbidity and mortality. Although it is recommended that establishing the medical home and family physician (FP) attachment prior to transfer will improve health outcomes, there is little evidence or policy surrounding the role of the FP during this transition. This study explores the described use of health services by adolescents with CHC/D’s, as well as the adolescent’s and caregiver’s perceptions of their FP.

**Methods:**

Participants were recruited from the British Columbia Children’s Hospital, Canada and a multi-method phased approach was used. In Phase 1, 84 adolescent and caregiver pairs completed questionnaires asking what medical services they accessed for specific health needs. In Phase 2, another cohort of 21 adolescent and caregiver pairs completed the same questionnaires and were interviewed regarding their perception of their FP.

**Results:**

96% (*n* = 81) of adolescents with CHC/D’s in phase 1 had a FP. Thirty four percent (*n* = 34) of adolescents had not seen their FP in the last 6 months. While adolescents with CHC/D mostly accessed their FP for primary health issues, they frequently also accessed specialists for prescription refills (50%, *n* = 51), mental health (29%, *n* = 30) and sexual health (16%, *n* = 16). While most adolescent/caregiver participants reported positive communication and trust in their FP, some had a poor understanding of the FP’s role in coordinating care.

**Conclusion:**

As many adolescents with CHC/D may see their FP infrequently and may not clearly understand their role, opportunities exist for strengthening primary care home attachment as well as adolescent and caregiver literacy around the potential contribution of the FP during and after transfer to adult services. Responsibility for improving care coordination for this population should be ideally shared between FPs and pediatric specialists.

**Electronic supplementary material:**

The online version of this article (10.1186/s12875-018-0830-6) contains supplementary material, which is available to authorized users.

## Background

An estimated 15–18% of youth in North America have a chronic health condition and/or disability (CHC/D) and over 98% of these youth are expected to reach age 20, thereby requiring transition out of a familiar, family-focused pediatric care system to a dramatically different adult care system where the family physician (FP) is the primary care provider [1]. However, adolescents often feel lost in the adult care system [2]. The adult medicine culture is less attenuated to the developmental complexities of adolescents and focuses on independence, making the transition from an integrated interdisciplinary pediatric system challenging [3]. Lack of engagement with the adult system is associated with lower rates of follow-up appointments and medical compliance [2, 4] and a higher risk of mortality and morbidity [5]. While the primary care doctor has a unique opportunity to address primary health care needs and facilitate the transition process through continuity of care [1, 6–8], much of the emphasis in the transition literature (which most likely reflects clinical practice) focuses solely on specialist-to-specialist provider transition [9, 10].

Young adults have suggested a number of strategies that may facilitate effective transition including peer support, mentorship, and having a health care professional who has knowledge and personal understanding of their condition oversee the transfer phase [11]. This concept aligns with the “medical home” model [6]. Implementing a medical home model where health care services are accessible, family centered, continuous, comprehensive, coordinated, and compassionate improves transition preparedness [12, 13]. The patient’s medical home can be defined as the place that patients feel most comfortable to discuss their health care, particularly for sensitive topics such as sexual health and mental health. It is a central hub to provide timely and coordinated care by an ongoing team of multidisciplinary caregivers, enabling best possible health outcomes [14]. There is evidence that a “shared care” model that includes patient engagement with specialists and FPs both prior to and after transfer results in better outcomes for adolescents with chronic health conditions such as diabetes [15].

In Canada, the medical home should be established at a FP’s practice as they are the first point of contact and primary coordinator of care [14]. However, not all pediatric patients have a FP as some general pediatricians provide on-going primary care in certain provinces. Patients may also access walk-in clinics staffed by different FPs, or emergency departments if they do not have a FP or if their FP is not available. Once a referral is made to a specialist, patients may continue to be followed by that specialist without the involvement of a FP. Although establishing a medical home with a primary care team leads to more effective transition planning, transition protocols often fail to adequately consider the role of the FP in long term management of adolescents following transition [16]. There is little empiric evidence to guide practice or policy surrounding the role of the primary health provider during this transition [1].

The patient-FP relationship is mediated by a variety of factors including the complexity of illness, inclusive management of the patient/family at a tertiary hospital, geography and accessibility to FPs, the perceived role and importance of the FP in continuing care, and the ability to find an FP [17, 18]. Furthermore, adolescents with CHC/Ds and their families may choose walk-in clinics, community pediatricians, the internet, an alternative health provider, or a member of their subspecialty team for interim health care management between necessary specialist appointments [19]. Although these may be convenient resources, lack of continuity prevents the establishment of the medical home at the FP’s office [20]. Another consideration regarding the patient-FP relationship unique to the transition period is the relationship between the adolescent and their caregiver with respect to health care management and decision-making, such as arranging appointments or seeing the physician alone during appointments. As adolescent patients transfer from pediatric family-centered care to autonomous adult care, the role of their caregiver in care coordination and management transforms. During this period, the adolescent and caregiver can be considered a decision-making unit in transition [21].

This study investigates adolescents with CHC/Ds′ described use of health care services and the adolescent and caregiver’s perspectives on the FP’s role in health care management prior to transfer to adult services. The goal is to inform strategies to improve the continuity of care with FPs through transition and transfer. We used descriptive and qualitative methods to investigate adolescent patients’ attachment with their FP by asking if they and their caregivers identified a FP, how often they saw them, and which health services they accessed for specific primary care issues. We also sought to determine the adolescent/caregiver’s perceived role of FPs in regular ongoing management and sentiments surrounding trust, knowledge, and overall relationship with their FP.

## Methods

This study was conducted in two phases using mixed methodology at the British Columbia Children’s Hospital (BCCH) in Vancouver, Canada. Phase 1 was conducted in 2013 and involved an online questionnaire that included multiple choice and open-ended responses. Phase 2 was conducted in 2014 and included the same questionnaire, with the addition of a qualitative face-to-face interview component that was informed from the open-ended data gathered from phase 1. The University of British Columbia Research Ethics Board approved the study and informed consent was obtained from all participants.

In Phase 1, a convenience sample (*n* = 84) of adolescent patient/caregiver pairs were invited to participate from the in-patient wards and during subspecialty clinic appointments at the BCCH. The adolescents and caregivers completed individual, parallel online questionnaires asking if they had a FP and where they would seek medical care for specific health issues (Additional file 1 and Additional file 2). Information on demographics (age, gender, geographic region), number of hospitalizations and number of specialists involved was also collected. We defined a subset population as medically complex if two or more subspecialty clinics were involved in the adolescent’s care, and/or the adolescent had at least 1 hospital admission in the past 12 months. The questionnaire asked participants about their relationship with their FP with respect to patient/provider communication, communication between providers, provider knowledge and trust. Concept domains to assess the FP’s involvement in care included the perceived role of the FP in routine care, the value of the FP’s role in context of multiple providers, and access to an FP. The questionnaire was developed by a team of FPs, pediatric/adult specialists, and the Transition Steering Committee of the Shared Care Youth Transition project [8]. The questionnaire was at a grade 6 reading level and took about 15 min to complete.

For Phase 2, we recruited a further convenience sample (*n* = 21) of adolescent inpatients/ clinic attendees and their caregivers who identified as having a FP. In addition to completing the questionnaire from Phase 1, we interviewed these adolescents and caregiver attendees about their experiences with their FP. The individual responses were recorded in the transcriptions.

The interview questions focused on adolescents’ and caregivers’ perspectives of communication and role of the FP in their care (Additional file 3). The interviews were 30 min long on average and were audiotaped and transcribed. Analysis was done using a qualitative description approach [22]. We used constant comparative analysis to derive thematic patterns within the interviews [23]. Coded themes were then independently reviewed and analyzed by two members of the research team who then discussed the themes to reach consensus.

### Inclusion and exclusion criteria

English speaking adolescents 14–18 years of age with a CHC/D who required transition to specialist adult care were included, with their caregivers if present. Caregivers who attended with adolescents with cognitive impairment were included even though the adolescent patient could not directly participate in the study. In phase 2, adolescents and their caregivers were excluded if they did not have a FP.

We compared the data sets from the parallel questionnaires completed independently by the adolescent patients and their caregivers and determined there was little variance between the data. However, the caregiver responses were more complete compared to the adolescent responses where questions were often left unanswered. In these cases, we determined that the caregiver represented the most competent coordinator of care and used this single data set of participants for analysis. When adolescents attended the clinic independently or when there were caregiver language barriers, the adolescent data was used for analysis. During the interviews in Phase 2, adolescents contributed equally so therefore both adolescent and caregiver responses were used for qualitative analysis. Data collected from adolescents and their caregivers in phase 1 was excluded from analysis if they indicated they did not have a FP.

## Results

In Phase 1, 84 adolescent-caregiver pairs were recruited between June–August 2014 with a response rate of 90%. Three pairs indicated not having a FP and were unable to answer questions surrounding access to and relationship with an FP. These pairs were excluded from further data analysis resulting in *n* = 81 for phase 1. In Phase 2, 21 pairs were recruited between June–August 2015 with a response rate of 67%. The main reason for refusing to participate in the study was lack of time. Data was collected from adolescent-caregiver pairs because most visits were attended by an adolescent patient with their caregiver. However, there were four adolescents with cognitive impairment who were not able to participate, and there were ten adolescents whose caregivers did not complete the questionnaire (either not present or language barriers). A total of 102 questionnaires were analyzed representing the most responsible coordinator of care from each pair (92 caregivers and 10 adolescents). In phase 2, 17 caregiver and 19 adolescent interview responses were subjected to qualitative analysis (4 caregivers and 2 adolescents could not complete the interview due to exclusion criteria).

### Demographics

The adolescents were 14–18 years old (mean 16.4 years) and 49% (*n* = 50) were 16–17 years old. Forty four percent (*n* = 45) of adolescent patients were male and 56% (*n* = 57) were female. Eighty three percent (*n* = 85) were from urban centers (within a 1 h drive of a regional hospital) (Additional file 4).

### Access to family physicians

In Phase 1, 96% (*n* = 81) of adolescents with CHC/D reported having a FP. These adolescents were asked if they ever see their FP independently. Only 3.75% (*n* = 3) indicated they always saw their FP alone. Sixty three percent (*n* = 51) always saw their FP accompanied by a parent/caregiver, while 28% (*n* = 23) occasionally saw their FP independently. Thirty eight percent (*n* = 39) had seen their FP two times or less over the past 2 years and 34% (*n* = 34) had not seen their FP in the last 6 months. A further 14% (*n* = 14) had not seen their FP in 12 months, and for 5% (*n* = 5), it was over 18 months since the last primary care visit.

### Accessing other medical services

FPs, hospital specialists, internet, alternative health providers, emergency room, and walk-in clinics were accessed by adolescent study participants in the past 12 months (Additional file 4). Most participants identified that they would see their FP exclusively for primary care issues such as immunizations (74%, *n* = 75), cold/flu like symptoms (68%, *n* = 69), injury (69%, *n* = 70), referrals (61%, *n* = 62), completion of forms (59%, *n* = 60). However, they were more likely to access specialists for prescription refills (50%, *n* = 51). While the FP was the sole source of care for 58% (*n* = 59) of adolescent patients for sexual health, and for 48% (*n* = 49) of adolescent patients for mental health, a substantial number would seek care from both FPs and specialists for sexual health (16%, *n* = 16) and mental health (29%, *n* = 30). Fifteen percent (*n* = 15) identified they would exclusively access the subspecialty clinic for mental health and 10% (*n* = 10) for sexual health. Regarding education about their medical condition, 36% (*n* = 37) of adolescent patients reported they would seek information from both FP and specialist, 29% (n = 30) from their specialist alone and 21% (*n* = 21) from their FP alone (Table 1).

**Table 1:** Physician services participants would access for specific care needs

Care needs	FP Only		BCCH subspecialty clinic only		Both		Neither	
Total *n* = 102	%	n	%	n	%	n	%	n
Medication Refill	40	41	25	26	25	25	10	10
Medication Side Effect	39	40	17	17	22	22	23	23
Education about medical condition	21	21	29	30	36	37	14	14
Sexual Health	58	59	10	10	6	6	26	27
Mental Health	48	49	15	15	14	14	24	24
Forms	59	60	10	10	15	15	17	17
Referral	61	62	8	8	24	24	8	8
Injury	69	70	0	0	11	11	21	21
Cold/flu like symptoms	68	69	3	3	10	10	20	20
Immunizations	74	75	0	0	6	6	21	21

Participants noted they would access other care services in addition or instead of consulting their FP or specialist (Table 2). Other services included the emergency room, walk-in clinics, alternative medicine, and the internet. The internet was selected as the primary source for education about their medical condition (31%, *n* = 32), sexual health (17%, *n* = 17) and mental health (17%, n = 17), while walk-in clinics were preferred for injuries (18%, *n* = 18), cold/flu like symptoms (14%, *n* = 14) and medication refills (10%, *n* = 10). In the interviews, five participants acknowledged that they obtained most of their care from walk-in clinics as their FP was not readily available, too far away, or too difficult to access. As per one caregiver: “Anything outside of his medical condition here, we would go to a walk-in clinic.”

**Table 2:** Other services participants would access for specific care needs

Care needs	Emergency Room		Walk in Clinic		Alternative Medicine		Internet		Other	
Total n = 102	%	n	%	n	%	n	%	n	%	n
Medication Refill	6	6	10	10	2	2	0	0	4	4
Medication Side Effect	15	15	9	9	2	2	9	9	7	7
Education about medical condition	2	2	2	2	4	4	31	32	4	4
Sexual Health	1	1	2	2	4	4	17	17	10	10
Mental Health	5	5	3	3	4	4	17	17	12	12
Forms	0	0	3	3	0	0	5	5	4	4
Referral	3	3	7	7	1	1	1	1	3	3
Injury	36	37	18	18	3	3	2	2	3	3
Cold/flu like symptoms	6	6	14	14	4	4	6	6	4	4
Immunizations	1	1	8	8	2	2	1	1	13	13

### Complex patients

Fifty two percent (*n* = 53) of adolescents were categorized as complex based on having 2 or more subspecialty clinics involved in their care and/or at least 1 hospital admission in the past 12 months. Compared to those who were less complex, complex adolescent patients seemed more likely to see their hospital specialist rather than their FP for sexual health (16% vs. 10%) and for mental health issues (24% vs. 14%). In the interviews, 4 adolescent/caregiver pairs reported that the majority of their care was with a specialist because the main concern was the significant health issue. As one pair explained, the main health concerns were “above and beyond our family doctor’s capabilities” such that “the family doctor could not handle it.”

### Relationship with FPs

The majority (59%, *n* = 60) of adolescents had the same FP for over ten years (Additional file 4). Most adolescent/caregiver participants shared the same family doctor (95%, *n* = 20), and agreed that their communication was generally good to excellent. They depicted communication as being conducive to sharing information (according to one adolescent, “you could talk to him about anything really”) and FPs as personable (a caregiver said, “he jokes with him, which is good. It’s good to make that connection.”) However, the interview data also revealed communication difficulties (Table 3). One adolescent noted his FP was “pretty narrow minded sometimes” and another a caregiver reported, “sometimes we feel like they are just not listening.”

**Table 3:** Adolescents and caregivers’ relationship with FP

Questions regarding relationship with FP	Not at all		Slightly		Very		Extremely	
Total n = 102	%	n	%	n	%	n	%	n
How comfortable do you and your youth feel asking questions of their FP	5	5	45	46	42	43	7	7
How likely are you and your youth to recommend your youth’s FP	9	9	23	23	43	44	25	25
How knowledgeable do you and your youth feel your youth’s FP is about their medical condition	8	8	45	46	38	39	8	8
How helpful is your FP at explaining your youth’s medical condition	13	13	35	36	37	38	14	14
How easy is it for your youth to get to their FP’s office	11	11	26	27	41	42	21	21
How easy is it to schedule urgent appointments with your youth’s FP when your youth is ill	20	20	34	35	32	33	13	13

When asked to reflect in Phase 2 on the role of the FP as compared to the role of the specialists, most participants (*n* = 15) felt more comfortable with the FP and had a sense that this was their medical home, even though they indicated the FP was “not for specific health problems or questions.” They explained this by noting, “he is closer,” “we see him more often” and “I’ve known him longer.” For some participants, this sense of continuity and familiarity was a valued attribute. One adolescent explained, “sometimes I’ll see him in public and I’ll say hi, so definitely family doctor for me.” In contrast, 4 participants indicated feeling more comfortable with specialists as they were more knowledgeable and had made a major difference to their care. In one family, the caregiver preferred the care of the specialist while the adolescent preferred his family doctor**.**

### Communication

With respect to communication between their specialist and FP care providers, most participants felt comfortable that necessary documentation had been sent and received. In their experience, the appropriate lab results and reports had been available in their files “most of the time.” However, few had a clear understanding of whether or not this involved any direct verbal communication. Their responses were characterized by vague impressions such as “I believe they would,” “I could imagine,” or “I’m not really sure.” One adolescent expressed, “I’m not a psychic. I don’t know what they say”.

### Perceived knowledge of FP

When asked about the relative degree to which their health care providers were knowledgeable, most thought that while specialists and FPs had particular skillsets, the specialist possessed considerably more knowledge about their specific condition. One adolescent noted, “If the family doctor was knowledgeable enough I wouldn’t be here at this hospital,” and one caregiver explained, “I am most comfortable bypassing my family doctor and talking to the specialists.”

### Care coordination

The perspective of most of participants was that care coordination was primarily a matter of referral to specialists, and most (*n* = 14) felt their FP was doing this well. As one account illustrated, the participant felt the FP would not be able to fill out medical forms, but could arrange referrals, and acknowledged, “The parts he can do, he will do well.” However, in two cases, specialist care was coordinated independently of the FP. In another, the adolescent felt the FP was coordinating care, but the caregiver described herself as coordinating with the specialist. Thus the idea of care coordination did not seem well understood by these families.

When explicitly questioned about their trust of the FP to manage their care, most of the participants (*n* = 16) agreed that their FP was trustworthy. They expressed this as “she knows the family dynamic,” “she knows how I react to things,” “he would tell us everything,” or “she’s open.” However, for the remainder, there were notable hesitations, including concerns about the level of knowledge. One adolescent stated, “He isn’t helpful, particularly when I am in pain.”

In Phase 2, all but one participant felt that having an FP had improved their overall health, explaining this as having to do with the continuity of care and breadth of interest in factors affecting their health. “She knows him, found his problem to start with, and has medical records,” “she is mostly the reason I can get better,” and noting that, when seeing a specialist “all the other stuff doesn’t get talked about.” Many aspects of care were more accessible with a FP than a specialist.

## Discussion

As most of the adolescent patients arrived at subspecialty appointments with their caregiver and did not see the specialist alone during the clinic visit, we determined that during the transition-planning period (i.e. prior to transfer to adult care), caregivers were particularly influential in determining where and whom they sought for care. However, during the interviews every adolescent could describe their relationship with their FP, giving their impressions regarding care management and the role of their physicians. This finding suggests that with appropriate support and encouragement, adolescent patients could be involved in developing independent decision making for their own health care, an important component of self-management.

Most participants identified a long-term relationship with a FP, listing continuity, familiarity and proximity to be valued attributes of their FP. Overall, adolescent patients and caregivers appreciated the contributions their FPs could make and recognized the distinction between the skillset and focus of the FP and the specialist. The majority had a positive relationship with the FP, feeling they were trustworthy and improved the adolescent’s overall health. Communications with their FP and between specialists and FPs were generally perceived as good, although few fully understood the intended alignment between the generalist and specialist systems. Several felt the FP’s role of coordinating medical care was primarily a matter of referral to specialists.

Despite the positive recognition of the role of the FP, there was a concerning lack of regular involvement of the FP in the adolescent’s medical care as determined by the number of reported patient visits in the previous two years. The most cited reason was that the FP was unavailable, too far, or too difficult to access, resulting in some participants choosing walk-in clinics for primary care. With increasing waiting times and difficulty in getting an appointment, patients are often turning to walk-in clinics. However, recently recommended practice management strategies designed to increase efficacy in the medical home model may mitigate this issue [24].

In some cases participants identified specialists as their source for primary health care, particularly when the adolescent patients had medically complex conditions (53% of our sample). These participants seem most likely to view their specialist as the exclusive care provider for concerns such as mental and sexual health. Some participants felt that the adolescent patient’s medical conditions were beyond the FP’s capabilities and therefore went directly to the specialist for medical concerns, establishing their medical home at the subspecialty clinic rather than with their FP. Not only is this an ineffective use of health care resources, it creates missed opportunities for establishing the FP’s office as the adolescent’s medical home [14]. When the specialist provides primary health care it creates a more significant handover at the time of transfer to adult specialist services. The FP who is unfamiliar with the patient needs to rapidly acquire the knowledge of a complex, chronic condition and develop a new patient/provider relationship to assume the role as the most responsible physician. Transfer to an entirely new set of providers is more likely to exacerbate the fear and resistance to transfer [25]. This is a significant concern as such adolescents are at higher risk for medical complications if lost to follow up care after transfer [5].

Given the paucity of evidence-based literature to guide practice or policy surrounding the role of the primary health provider during transition to adult services, there are some limitations in this novel study. The study was based on a small sample size, especially when narrowed down to more complex patients. A larger sample size would benefit future investigations to determine if there is a difference in the attachment to the FP between complex and non-complex adolescents, and where they establish their medical home prior to transition. Furthermore, participants were recruited via a convenience sample from multiple clinics and therefore our sample was not randomized. The data used for analysis represented the most competent coordinator of care, which was the caregiver in most cases except when the adolescent attended the clinic alone or when there were caregiver language barriers. Some questions in the youth questionnaires were left unanswered, and this may have been due to lack of knowledge or engagement with the questionnaire. Obtaining a complete and reliable set of adolescent responses for analysis would be valuable in highlighting the adolescents’ use of the internet, walk-in clinics and youth clinics for medical care, particularly for sensitive topics such as mental and sexual health. Using a shorter, simplified, youth-friendly questionnaire with images designed to engage adolescents could yield such results in future studies.

## Conclusions

There is limited empiric evidence to guide primary care interventions to improve transition outcomes for adolescents with CHC/D’s [1]. As a result of this study, we believe that opportunities exist for strengthening the attachment adolescents with CHC/Ds have with their FP as the primary manager of care. As the FP becomes the unequivocal most responsible physician after transfer, facilitating a strong FP-patient relationship and establishing the medical home results in a more effective transition [12, 13]. Given that only 3.75% of adolescents with CHC/D’s regularly see their FP alone, health care providers and caregivers should encourage adolescents to become more independent in the management of their own health care. Preparation for transition should start early in childhood [26] so that they experience a graded responsibility that facilitates their interactions with the health care system separate from that of their caregivers.

Responsibility for improving adolescent patient/caregiver health literacy and helping them understand the role of the FP in the adolescent’s health management should be shared between FPs and pediatric specialists. In order to ensure continuity of care past the pediatric age, specialists have an opportunity to facilitate the relationship with their FP by encouraging adolescents to see their FP for primary health issues in between specialist visits. With support from pediatric specialists, including clear communication as to the needs of these special populations, FPs can play an integral role in improving health outcomes for adolescents with CHC/Ds during transition.

## Additional files

Additional file 1: Caregiver Questionnaire Questionnaire completed by caregivers of adolescent patients. (DOCX 92 kb)

Additional file 2: Youth Questionnaire Questionnaire completed by adolescent patients. (DOCX 92 kb)

Additional file 3: Extension interview questions These interview questions focused on adolescents’ and caregivers’ perspectives of communication and role of the FP in their care. (DOCX 77 kb)

Additional file 4: Appendix This appendix includes supplemental information regarding patient demographics, access to medical services, and relationship with the FP. (DOCX 47 kb)

## Abbreviations

CHC/D: Chronic health condition and/or disability
FP: Family physician
