# Supplementary material for: Perceptions of the family physician from adolescents and their caregivers preparing to transition to adult care
Source: BMC Fam Pract. 2018 Aug 23;19:140. doi: 10.1186/s12875-018-0830-6 (PMC6106717; doi:10.1186/s12875-018-0830-6)
Supplement: Supplementary file 1 — Caregiver Questionnaire Questionnaire completed by caregivers of adolescent patients. (DOCX 92 kb) [file 12875_2018_830_MOESM1_ESM.docx]

**FP Study Caregiver Questionnaire**

1. Please select the area where you and your youth have been approached to complete this survey.
   - BC Children’s Hospital ward
   - BC Children’s Hospital neurology clinic
   - BC Children’s Hospital cardiology clinic
   - BC Children’s Hospital renal clinic
   - BC Children’s Hospital GI clinic
   - BC Children’s Hospital endocrine/diabetes clinic
2. Is your youth male or female?
   - Male
   - Female
3. Year of survey
4. What is your youth's year of birth?
5. What is your youth’s age?
6. What city do you currently live in?
7. Do you live in an urban community (within one hour drive of regional hospital) ?
8. What health authority do you belong to?
   - VIHA (Vancouver Island Health Authority)
   - IHA (Interior Health Authority)
   - VCH (Vancouver Coastal Health Authority)
   - FHA (Fraser Health Authority)
   - NHA (Northern Health Authority)
   - Yukon Health Authority
9. How many specialty clinics is your youth followed by at BC Children's Hospital (BCCH)?
   - 1
   - 2
   - 3
   - 4
   - 5+
10. How many times has your youth visited your BC Children's Hospital Specialist in the past 12 months?
    - 1
    - 2
    - 3+
11. In addition to your specialty clinic(s) at BC Children's Hospital, does your youth have a regular paediatrician?
    - Yes
    - No
12. Has your youth required a hospital admission in the past 12 months?
    - No
    - Yes, just one
    - Yes, multiple
13. Which medical services have you used for your youth in the past 12 months? (check all that apply)
    - Family Doctor
    - Pediatrician
    - BC Childrens Hospital Specialty Clinic (Physician, Nurse)
    - Hospital Emergency Department (Community or BC Childrens)
    - Walk-in Clinic
    - Alternative Medicine (ie Naturopath, Chinese Medicine)
    - Internet (ie Google)
    - Youth Clinic
    - Other (Please specify)
14. Please select the health care services you would access for your youth for **medication refill** (check all that apply)
    - Family Doctor
    - Pediatrician
    - BC Childrens Hospital Specialty Clinic (Physician, Nurse)
    - Hospital Emergency Department (Community or BC Childrens)
    - Walk-in Clinic
    - Alternative Medicine (ie Naturopath, Chinese Medicine)
    - Internet (ie Google)
    - Youth Clinic
    - Other (Please specify)
15. Please select the health care services you would access for your youth for **medication side effects or allergic reaction** (check all that apply)
    - Family Doctor
    - Pediatrician
    - BC Childrens Hospital Specialty Clinic (Physician, Nurse)
    - Hospital Emergency Department (Community or BC Childrens)
    - Walk-in Clinic
    - Alternative Medicine (ie Naturopath, Chinese Medicine)
    - Internet (ie Google)
    - Youth Clinic
    - Other (Please specify)
16. Please select the health care services you would access for your youth for **education about your youth’s medical condition** (check all that apply)
    - Family Doctor
    - Pediatrician
    - BC Childrens Hospital Specialty Clinic (Physician, Nurse)
    - Hospital Emergency Department (Community or BC Childrens)
    - Walk-in Clinic
    - Alternative Medicine (ie Naturopath, Chinese Medicine)
    - Internet (ie Google)
    - Youth Clinic
    - Other (Please specify)
17. Please select the health care services you would access for your youth for **sexual health (ie. education, development, birth control)** (check all that apply)
    - Family Doctor
    - Pediatrician
    - BC Childrens Hospital Specialty Clinic (Physician, Nurse)
    - Hospital Emergency Department (Community or BC Childrens)
    - Walk-in Clinic
    - Alternative Medicine (ie Naturopath, Chinese Medicine)
    - Internet (ie Google)
    - Youth Clinic
    - Other (Please specify)
18. Please select the health care services you would access for your youth for **mental health (feeling sad, anxious, stressed)** (check all that apply)
    - Family Doctor
    - Pediatrician
    - BC Childrens Hospital Specialty Clinic (Physician, Nurse)
    - Hospital Emergency Department (Community or BC Childrens)
    - Walk-in Clinic
    - Alternative Medicine (ie Naturopath, Chinese Medicine)
    - Internet (ie Google)
    - Youth Clinic
    - Other (Please specify)
19. Please select the health care services you would access for your youth for **forms to be completed (ie. insurance or driver's form)** (check all that apply)
    - Family Doctor
    - Pediatrician
    - BC Childrens Hospital Specialty Clinic (Physician, Nurse)
    - Hospital Emergency Department (Community or BC Childrens)
    - Walk-in Clinic
    - Alternative Medicine (ie Naturopath, Chinese Medicine)
    - Internet (ie Google)
    - Youth Clinic
    - Other (Please specify)
20. Please select the health care services you would access for your youth for **ordering a test or procedure (ie. blood work, ultrasound)** (check all that apply)
    - Family Doctor
    - Pediatrician
    - BC Childrens Hospital Specialty Clinic (Physician, Nurse)
    - Hospital Emergency Department (Community or BC Childrens)
    - Walk-in Clinic
    - Alternative Medicine (ie Naturopath, Chinese Medicine)
    - Internet (ie Google)
    - Youth Clinic
    - Other (Please specify)
21. Please select the health care services you would access for your youth for a **referral (ie. to a specialist doctor, physiotherapy or massage)** (check all that apply)
    - Family Doctor
    - Pediatrician
    - BC Childrens Hospital Specialty Clinic (Physician, Nurse)
    - Hospital Emergency Department (Community or BC Childrens)
    - Walk-in Clinic
    - Alternative Medicine (ie Naturopath, Chinese Medicine)
    - Internet (ie Google)
    - Youth Clinic
    - Other (Please specify)
22. Please select the health care services you would access for your youth for an **injury** (check all that apply)
    - Family Doctor
    - Pediatrician
    - BC Childrens Hospital Specialty Clinic (Physician, Nurse)
    - Hospital Emergency Department (Community or BC Childrens)
    - Walk-in Clinic
    - Alternative Medicine (ie Naturopath, Chinese Medicine)
    - Internet (ie Google)
    - Youth Clinic
    - Other (Please specify)
23. Please select the health care services you would access for your youth for **cold/flu like symptoms** (check all that apply)
    - Family Doctor
    - Pediatrician
    - BC Childrens Hospital Specialty Clinic (Physician, Nurse)
    - Hospital Emergency Department (Community or BC Childrens)
    - Walk-in Clinic
    - Alternative Medicine (ie Naturopath, Chinese Medicine)
    - Internet (ie Google)
    - Youth Clinic
    - Other (Please specify)
24. Please select the health care services you would access for your youth for **immunizations** (check all that apply)
    - Family Doctor
    - Pediatrician
    - BC Childrens Hospital Specialty Clinic (Physician, Nurse)
    - Hospital Emergency Department (Community or BC Childrens)
    - Walk-in Clinic
    - Alternative Medicine (ie Naturopath, Chinese Medicine)
    - Internet (ie Google)
    - Youth Clinic
    - Other (Please specify)
25. Please select the health care services you would access for your youth for **education about your youths medical condition** (check all that apply)
    - Family Doctor
    - Pediatrician
    - BC Childrens Hospital Specialty Clinic (Physician, Nurse)
    - Hospital Emergency Department (Community or BC Childrens)
    - Walk-in Clinic
    - Alternative Medicine (ie Naturopath, Chinese Medicine)
    - Internet (ie Google)
    - Youth Clinic
    - Other (Please specify)
26. Please select the health care services you would access for your youth for **education about your youths medical condition** (check all that apply)
    - Family Doctor
    - Pediatrician
    - BC Childrens Hospital Specialty Clinic (Physician, Nurse)
    - Hospital Emergency Department (Community or BC Childrens)
    - Walk-in Clinic
    - Alternative Medicine (ie Naturopath, Chinese Medicine)
    - Internet (ie Google)
    - Youth Clinic
    - Other (Please specify)
27. Does your youth you have a Family Doctor?
    - If not, why does your youth not have a family doctor?
      - Can’t find one
      - I don’t like the one my family uses
      - I have just moved to the area
      - I do not need a family doctor
28. How did you/your youth find their Family Doctor?
29. How long has your youth had their Family Doctor?
30. When was the last time your youth had an appointment with their Family Doctor?
31. How long has your youth had their Family Doctor?
    - Less than 2 years
    - 2-5 years
    - 6-10 years
    - 10+ years
32. When was the last time your youth had an appointment with their Family Doctor?
    - Less than one month
    - 1-6 months
    - 7-12 months
    - 12-18 months
    - greater than 18 months
33. How often has your youth seen their Family Doctor in the past 2 years?
    - Never
    - Once
    - Twice
    - Three or more
34. How comfortable does your youth feel asking questions of their Family Doctor?
    - Not at all comfortable
    - Slightly comfortable
    - Very comfortable
    - Extremely comfortable
35. How likely are you to recommend your youth's Family Doctor to family or friends?
    - Not at all likely
    - Slightly likely
    - Very likely
    - Extremely likely
36. During a typical office visit, does your youth’s Family Doctor spend too much time with you, too little time with you, or about the right amount of time with you?
    - Slightly too little
    - About the right amount
    - Slightly too much
37. How knowledgeable do you feel your youth's Family Doctor is about their medical condition?
    - Not at all knowledgeable
    - Slightly knowledgeable
    - Very knowledgeable
    - Extremely knowledgeable
38. How helpful is your Family Doctor at explaining your youth's medical condition(s)?
    - Not at all helpful
    - Slightly helpful
    - Very helpful
    - Extremely helpful
39. How much do you trust your Family Doctor to make decisions that are in your youth's best interests?
    - Not at all
    - A little
    - A lot
    - A great deal
40. How easy is it for your youth to get to their Family Doctor's office?
    - Not at all easy
    - Slightly easy
    - Very easy
    - Extremely easy
41. How easy is it to schedule urgent appointments with your youth's Family Doctor when your youth is ill?
    - Not at all easy
    - Slightly easy
    - Very easy
    - Extremely easy
42. Do you feel there is good communication between your youth's Family Doctor and their specialist team?
    - No
    - Not sure
    - Yes
43. Overall, how satisfied are you with your Family Doctor?
    - Very dissatisfied
    - Moderately satisfied
    - Very satisfied
44. How well does your youth's Family Doctor listen to you and your youth?
    - Not at all well
    - Slightly well
    - Moderately well
    - Very well
45. How well does your Family Doctor answer yours and your youth's questions?
    - Not at all well
    - Slightly well
    - Moderately well
    - Very well
46. Please add any comments you may have as to 'why' or 'why not' you feel your Family Doctor is an important member of your youth’s health care team.
