# Supplementary material for: Perceptions of the family physician from adolescents and their caregivers preparing to transition to adult care
Source: BMC Fam Pract. 2018 Aug 23;19:140. doi: 10.1186/s12875-018-0830-6 (PMC6106717; doi:10.1186/s12875-018-0830-6)
Supplement: Supplementary file 2 — Youth Questionnaire Questionnaire completed by adolescent patients. (DOCX 92 kb) [file 12875_2018_830_MOESM2_ESM.docx]

**FP Study Youth Questionnaire**

1. Please select the area where you have been approached to complete this survey.
   - BC Children’s Hospital ward
   - BC Children’s Hospital neurology clinic
   - BC Children’s Hospital cardiology clinic
   - BC Children’s Hospital renal clinic
   - BC Children’s Hospital GI clinic
   - BC Children’s Hospital endocrine/diabetes clinic
2. Are you male or female?
   - Male
   - Female
3. Year of survey
4. What is your year of birth?
5. What is your age?
6. What city do you currently live in?
7. Do you live in an urban community (within one hour drive of regional hospital)?
8. What health authority do you belong to?
   - VIHA (Vancouver Island Health Authority)
   - IHA (Interior Health Authority)
   - VCH (Vancouver Coastal Health Authority)
   - FHA (Fraser Health Authority)
   - NHA (Northern Health Authority)
   - Yukon Health Authority
9. How many specialty clinics are you followed by at BC Children's Hospital (BCCH)?
   - 1
   - 2
   - 3
   - 4
   - 5+
10. How many times have you visited your BC Children's Hospital Specialist in the past 12 months?
    - 1
    - 2
    - 3+
11. In addition to your specialty clinic(s) at BC Children's Hospital, do you have a regular paediatrician?
    - Yes
    - No
12. Have you required a hospital admission in the past 12 months?
    - No
    - Yes, just one
    - Yes, multiple
13. Which medical services have you used in the past 12 months? (check all that apply)
    - Family Doctor
    - Pediatrician
    - BC Childrens Hospital Specialty Clinic (Physician, Nurse)
    - Hospital Emergency Department (Community or BC Childrens)
    - Walk-in Clinic
    - Alternative Medicine (ie Naturopath, Chinese Medicine)
    - Internet (ie Google)
    - Youth Clinic
    - Other (Please specify)
14. Please select the health care services you would access for **medication refill** (check all that apply)
    - Family Doctor
    - Pediatrician
    - BC Childrens Hospital Specialty Clinic (Physician, Nurse)
    - Hospital Emergency Department (Community or BC Childrens)
    - Walk-in Clinic
    - Alternative Medicine (ie Naturopath, Chinese Medicine)
    - Internet (ie Google)
    - Youth Clinic
    - Other (Please specify)
15. Please select the health care services you would access for **medication side effects or allergic reaction** (check all that apply)
    - Family Doctor
    - Pediatrician
    - BC Childrens Hospital Specialty Clinic (Physician, Nurse)
    - Hospital Emergency Department (Community or BC Childrens)
    - Walk-in Clinic
    - Alternative Medicine (ie Naturopath, Chinese Medicine)
    - Internet (ie Google)
    - Youth Clinic
    - Other (Please specify)
16. Please select the health care services you would access for **education about your youth’s medical condition** (check all that apply)
    - Family Doctor
    - Pediatrician
    - BC Childrens Hospital Specialty Clinic (Physician, Nurse)
    - Hospital Emergency Department (Community or BC Childrens)
    - Walk-in Clinic
    - Alternative Medicine (ie Naturopath, Chinese Medicine)
    - Internet (ie Google)
    - Youth Clinic
    - Other (Please specify)
17. Please select the health care services you would access for **sexual health (ie. education, development, birth control)** (check all that apply)
    - Family Doctor
    - Pediatrician
    - BC Childrens Hospital Specialty Clinic (Physician, Nurse)
    - Hospital Emergency Department (Community or BC Childrens)
    - Walk-in Clinic
    - Alternative Medicine (ie Naturopath, Chinese Medicine)
    - Internet (ie Google)
    - Youth Clinic
    - Other (Please specify)
18. Please select the health care services you would access for **mental health (feeling sad, anxious, stressed)** (check all that apply)
    - Family Doctor
    - Pediatrician
    - BC Childrens Hospital Specialty Clinic (Physician, Nurse)
    - Hospital Emergency Department (Community or BC Childrens)
    - Walk-in Clinic
    - Alternative Medicine (ie Naturopath, Chinese Medicine)
    - Internet (ie Google)
    - Youth Clinic
    - Other (Please specify)
19. Please select the health care services you would access for **forms to be completed (ie. insurance or driver's form)** (check all that apply)
    - Family Doctor
    - Pediatrician
    - BC Childrens Hospital Specialty Clinic (Physician, Nurse)
    - Hospital Emergency Department (Community or BC Childrens)
    - Walk-in Clinic
    - Alternative Medicine (ie Naturopath, Chinese Medicine)
    - Internet (ie Google)
    - Youth Clinic
    - Other (Please specify)
20. Please select the health care services you would access for **ordering a test or procedure (ie. blood work, ultrasound)** (check all that apply)
    - Family Doctor
    - Pediatrician
    - BC Childrens Hospital Specialty Clinic (Physician, Nurse)
    - Hospital Emergency Department (Community or BC Childrens)
    - Walk-in Clinic
    - Alternative Medicine (ie Naturopath, Chinese Medicine)
    - Internet (ie Google)
    - Youth Clinic
    - Other (Please specify)
21. Please select the health care services you would access for a **referral (ie. to a specialist doctor, physiotherapy or massage)** (check all that apply)
    - Family Doctor
    - Pediatrician
    - BC Childrens Hospital Specialty Clinic (Physician, Nurse)
    - Hospital Emergency Department (Community or BC Childrens)
    - Walk-in Clinic
    - Alternative Medicine (ie Naturopath, Chinese Medicine)
    - Internet (ie Google)
    - Youth Clinic
    - Other (Please specify)
22. Please select the health care services you would access for an **injury** (check all that apply)
    - Family Doctor
    - Pediatrician
    - BC Childrens Hospital Specialty Clinic (Physician, Nurse)
    - Hospital Emergency Department (Community or BC Childrens)
    - Walk-in Clinic
    - Alternative Medicine (ie Naturopath, Chinese Medicine)
    - Internet (ie Google)
    - Youth Clinic
    - Other (Please specify)
23. Please select the health care services you would access for **cold/flu like symptoms** (check all that apply)
    - Family Doctor
    - Pediatrician
    - BC Childrens Hospital Specialty Clinic (Physician, Nurse)
    - Hospital Emergency Department (Community or BC Childrens)
    - Walk-in Clinic
    - Alternative Medicine (ie Naturopath, Chinese Medicine)
    - Internet (ie Google)
    - Youth Clinic
    - Other (Please specify)
24. Please select the health care services you would access for **immunizations** (check all that apply)
    - Family Doctor
    - Pediatrician
    - BC Childrens Hospital Specialty Clinic (Physician, Nurse)
    - Hospital Emergency Department (Community or BC Childrens)
    - Walk-in Clinic
    - Alternative Medicine (ie Naturopath, Chinese Medicine)
    - Internet (ie Google)
    - Youth Clinic
    - Other (Please specify)
25. Please select the health care services you would access for **education about your youths medical condition** (check all that apply)
    - Family Doctor
    - Pediatrician
    - BC Childrens Hospital Specialty Clinic (Physician, Nurse)
    - Hospital Emergency Department (Community or BC Childrens)
    - Walk-in Clinic
    - Alternative Medicine (ie Naturopath, Chinese Medicine)
    - Internet (ie Google)
    - Youth Clinic
    - Other (Please specify)
26. Please select the health care services you would access for **education about your youths medical condition** (check all that apply)
    - Family Doctor
    - Pediatrician
    - BC Childrens Hospital Specialty Clinic (Physician, Nurse)
    - Hospital Emergency Department (Community or BC Childrens)
    - Walk-in Clinic
    - Alternative Medicine (ie Naturopath, Chinese Medicine)
    - Internet (ie Google)
    - Youth Clinic
    - Other (Please specify)
27. Do you have a Family Doctor?
    - If not, why do you not have a family doctor?
      - Can’t find one
      - I don’t like the one my family uses
      - I have just moved to the area
      - I do not need a family doctor
28. How did you find your Family Doctor?
29. How long have you had your Family Doctor?
30. When was the last time you had an appointment with your Family Doctor?
31. How long have you had your Family Doctor?
    - Less than 2 years
    - 2-5 years
    - 6-10 years
    - 10+ years
32. When was the last time you had an appointment with your Family Doctor?
    - Less than one month
    - 1-6 months
    - 7-12 months
    - 12-18 months
    - greater than 18 months
33. How often have you seen your Family Doctor in the past 2 years?
    - Never
    - Once
    - Twice
    - Three or more
34. How comfortable do you feel asking questions of your Family Doctor?
    - Not at all comfortable
    - Slightly comfortable
    - Very comfortable
    - Extremely comfortable
35. How likely are you to recommend your Family Doctor to family or friends?
    - Not at all likely
    - Slightly likely
    - Very likely
    - Extremely likely
36. Do you ever see your Family Doctor without your parent/caregiver?
    - Never
    - Sometimes
    - Always
37. During a typical office visit, does your Family Doctor spend too much time with you, too little time with you, or about the right amount of time with you?
    - Slightly too little
    - About the right amount
    - Slightly too much
38. How knowledgeable do you feel your Family Doctor is about your medical condition?
    - Not at all knowledgeable
    - Slightly knowledgeable
    - Very knowledgeable
    - Extremely knowledgeable
39. How helpful is your Family Doctor at explaining your medical condition(s)?
    - Not at all helpful
    - Slightly helpful
    - Very helpful
    - Extremely helpful
40. How much do you trust your Family Doctor to make decisions that are in your best interests?
    - Not at all
    - A little
    - A lot
    - A great deal
41. How well do you feel your Family Doctor respects your privacy and maintains confidentiality?
    - Not very well
    - Moderately well
    - Very well
    - Extremely well
42. How easy is it for you to get to your Family Doctor's office?
    - Not at all easy
    - Slightly easy
    - Very easy
    - Extremely easy
43. How easy is it to schedule urgent appointments with your Family Doctor when you are ill?
    - Not at all easy
    - Slightly easy
    - Very easy
    - Extremely easy
44. Do you feel there is good communication between your Family Doctor and your specialist team?
    - No
    - Not sure
    - Yes
45. Overall, how satisfied are you with your Family Doctor?
    - Very dissatisfied
    - Moderately satisfied
    - Very satisfied
46. How well does your Family Doctor listen to you?
    - Not at all well
    - Slightly well
    - Moderately well
    - Very well
47. How well does your Family Doctor answer yours questions?
    - Not at all well
    - Slightly well
    - Moderately well
    - Very well
48. Please add any comments you may have as to 'why' or 'why not' you feel your Family Doctor is an important member of your health care team.
