# Supplementary material for: Perceptions of the family physician from adolescents and their caregivers preparing to transition to adult care
Source: BMC Fam Pract. 2018 Aug 23;19:140. doi: 10.1186/s12875-018-0830-6 (PMC6106717; doi:10.1186/s12875-018-0830-6)
Supplement: Supplementary file 3 — Extension interview questions These interview questions focused on adolescents’ and caregivers’ perspectives of communication and role of the FP in their care. (DOCX 77 kb) [file 12875_2018_830_MOESM3_ESM.docx]

**FP Study Extension Interview Questions**

1. You have indicated you have a regular family doctor.
   1. Is your family doctor the same as the rest of the family?
2. Do you and your family doctor communicate openly and honestly with one another about your health and care options?
3. Do you seek the majority of your care from your family doctor?
   1. Do you have a pediatrician?
   2. What do you see as the role of the family doctor as compared to the role of the specialists?
4. Please comment on the communication between your health care providers.
   1. Do they have the reports and information about you on hand when you attend an appointment?
   2. Do you request copies of your health reports?
5. Please comment on the knowledge of your health care providers
   1. Can your family doctor coordinate your care to address most of your health concerns?
   2. Please comment on your trust of the family doctor to manage your care.
6. Do you feel having a family doctor has improved your overall health?
7. Are you aware of the BC College of Family Physician’s website?
8. Do you have any other additional comments about your experiences with your family doctor?
