# Supplementary material for: Perceptions of the family physician from adolescents and their caregivers preparing to transition to adult care
Source: BMC Fam Pract. 2018 Aug 23;19:140. doi: 10.1186/s12875-018-0830-6 (PMC6106717; doi:10.1186/s12875-018-0830-6)
Supplement: Supplementary file 4 — Appendix This appendix includes supplemental information regarding patient demographics, access to medical services, and relationship with the FP. (DOCX 47 kb) [file 12875_2018_830_MOESM4_ESM.docx]

**Appendix**

**Patient demographics**

**Access to medical services**

**Relationship with FP**
